# Saunders’ Pocket Medical Formulary

**Published:** 1892-06

**Authors:** 


SAUNDERS' POCKET MEDICAL FORMULARY. With an Appendix, containing Posological Table, Formulæ and Doses for Hypodermic Medication; Poisons and their Antidotes; Diameters of the Female Pelvis and Fetal Head; Diet List for Various Diseases; Obstetrical Table; Materials and Drugs used in Antiseptic Surgery, etc. By WILLIAM M. POWELL, M. D., author of Essentials of Diseases of Children; one of the Associate Editors of the Annual of the Universal Medical Sciences; Attending Physician to the Children's Seashore House for Invalid Children, and the Mercer House for Invalid Women, at Atlantic City, N. J.; Member of the Philadelphia Pathological Society. Formerly Instructor of Physical Diagnosis in University of Pennsylvania; Attending Physician to the Children's Clinic at the University of St. Clement's Hospital; Chief of the Medical Clinic of the Philadelphia Polyclinic. Philadelphia: W. B. Saunders, 913 Walnut street. 1891.

Whoever desires to have a ready-made prescription book will find one of the best in this little work. We are not of those who believe that ready-made formulæ are of much value, or that they possess much merit in the literature of medicine, but as there is a demand for this kind of therapeutics, we are glad to have it done in good form and with some attention to accuracy. The authors quoted from for the most part are reliable, and this book, as we have remarked, fills the place that it has marked out for itself as well as or even better than some that have attempted to cover this field.

